# Specimens for Museum of Ohio College of Dental Surgeons

**Published:** 1851-10

**Authors:** 


Specimens for the Museum of the Ohio College of Dental Surgery.—We are glad the profession is not forgetting this collection of rarities.

We have received a specimen of Artificial Work, by Dr. H. E. Peebles, Lexington, Missouri.

A specimen of Osseous union of two Deciduous Teeth, presented by Dr. Scott, Lancaster—And a Mastadon's Tooth, by Dr. Ulrey, Rising Sun, Indiana.

We shall give these and some others a more special notice in next number.

We have been obliged to crowd out much matter we should like to have given in the present number.

